# BCAR-Net: A Bidirectional Cross-Attention Network with Auxiliary Reconstruction for Tree Counting in Complex Forest Scenes Using Airborne RGB and LiDAR Data

**DOI:** 10.3390/plants15121762

**Published:** 2026-06-06

**Authors:** Xiaoyu Wu, Xijian Fan, Mengjiao Tang, Size Dai

**Affiliations:** College of Information Science and Technology & Artificual Intelligence, Nanjing Forestry University, Longpan Road 159, Nanjing 210037, China; xiaoyuwu22@njfu.edu.cn (X.W.); tangmengjiao@njfu.edu.cn (M.T.); daisize@njfu.edu.cn (S.D.)

**Keywords:** individual tree counting, multimodal fusion, density map estimation, close-range remote sensing

## Abstract

Accurate tree counting from remote sensing data is essential for forest inventory, biomass estimation, carbon accounting, and ecological monitoring. However, existing approaches predominantly rely on airborne RGB imagery and often struggle in complex forest scenes where neighboring crowns exhibit highly similar textures and colors and where overlapping crown boundaries become ambiguous. To address this limitation, the LiDAR-derived Canopy Height Model (CHM) is introduced as a complementary modality that provides explicit cues on canopy height variation and vertical structure to support RGB-based analysis. Building on this, we propose BCAR-Net, a broker-guided RGB and depth (RGB-D) multimodal framework that couples bidirectional cross-modal interaction, adaptive tri-branch fusion, and auxiliary reconstruction within a two-stage optimization scheme. Specifically, a bidirectional cross-attention U-Net generates an intermediate broker RGB-D representation from paired RGB images and depth maps through symmetric bidirectional cross-attention between the two modalities and direction-aware gating. The original RGB image, depth map, and broker representation are then jointly encoded by three weight-sharing branches and adaptively aggregated by a spatial fusion gate for density-map regression. To regularize the fused latent feature, a multi-scale cross-attention reconstruction decoder provides auxiliary RGB and depth reconstruction supervision by querying multi-scale BCA-UNet encoder features through 2D cross-attention, and a reconstruction-oriented first stage replaces externally generated fused-image supervision, yielding a task-consistent optimization scheme. Experiments on the NEONTreeEvaluation benchmark show that BCAR-Net consistently outperforms single-modality settings and direct RGB-D concatenation multimodal baseline. Additional experiments on a public UAV RGB–LiDAR dataset provide a small-scale supplementary evaluation under a different acquisition setting, where BCAR-Net achieves modest but consistent improvements over RGB-only and depth-only baselines. These results demonstrate that the proposed framework offers an effective but computationally cautious solution for tree counting in complex forest environments.

## 1. Introduction

Accurate tree counting from remote sensing data underpins a wide range of forest and ecological applications, including forest inventory, aboveground biomass and carbon-stock estimation, biodiversity monitoring, and the management of trees outside forest [1,2,3,4]. Recent continental-scale studies have demonstrated that wall-to-wall mapping and counting of individual trees can quantify carbon stocks in dryland ecosystems [2], reveal substantial tree cover outside areas previously classified as forest [5], and support tree-level monitoring at national scale [4], fundamentally reshaping the spatial granularity of forest assessment. With the rapid growth of high-resolution airborne and UAV remote sensing, automated tree counting has therefore become a foundational task at the intersection of computer vision, photogrammetry and ecological monitoring [6,7].

Existing approaches to quantifying trees from remote sensing imagery can be broadly divided into object detection-based and density map-based formulations. Object detectors such as Faster R-CNN, YOLO and RetinaNet [8,9,10] predict an explicit bounding box for every instance, but their performance degrades sharply in dense, occluded, and heavily overlapping scenes: heavy crown overlap inflates the intersection-over-union among true positives, non-maximum suppression then suppresses correct detections together with duplicates, and box-level regression provides little spatial information about how instances are distributed within an image [11,12]. As an alternative, density map regression treats counting as the prediction of a per-pixel object-density field whose spatial integral equals the object count and supervises the network with simple point or box-derived density targets rather than precise bounding box coordinates [13,14,15]. Because density maps avoid explicit instance separation, they are inherently more robust to severe occlusion and inter-object overlap, while still preserving the spatial distribution of objects [11]. This advantage has been repeatedly confirmed in agricultural counting tasks, where canopies and organs are densely packed and frequently mutually occluded. The TasselNet family demonstrated that local-count regression can count maize tassels and wheat ears at densities that defeat box-based detectors, with TasselNetV2+ outperforming Faster R-CNN on multiple in-field crop datasets [16,17,18]. Subsequent work has extended this paradigm to wheat head counting under heavy leaf occlusion [19] and UAV-based maize stand counting [20]. In remote sensing, density map counting has likewise proven effective for plantation and individual tree counting from UAV and satellite imagery [12,21,22], with VrsNet explicitly arguing that bounding-box-based methods have struggled to convey semantic information about tree crowns in closed-canopy UAV scenes [12]. Motivated by this evidence, the present work formulates tree counting as density-map regression rather than object detection, which is better aligned with the dense overlapping canopies that dominate complex forest scenes.

Within the image-based stream, RGB imagery has been by far the most extensively explored modality for tree analysis from remote sensing, owing to its high spatial resolution and rich texture and color information. While the present work targets tree-count estimation rather than individual crown delineation, related RGB-based studies cover the broader span of individual-tree detection, crown segmentation, and crown mapping. Representative approaches include the widely used DeepForest framework, which couples a RetinaNet detector with semi-supervised pretraining for tree-crown delineation in aerial RGB images [23]; Mask R-CNN-based instance segmentation systems such as Detectree2 for tropical forest crowns [24]; uncertainty-aware single-stage detectors such as TCDNet [25]; and density-regression UAV counters such as VrsNet [12]. National- and continental-scale studies have further shown that RGB deep learning can support tree counting, crown segmentation, and height estimation at large scale [1,26].

Despite these advances, RGB-only methods exhibit well-documented limitations in complex forest scenes. Color and texture become unreliable in shadowed regions, where the spectral signature deviates substantially from sunlit canopies and reduces classification accuracy [27]; spectrally similar species and visually similar crowns are difficult to disambiguate from appearance cues alone [7,27]; and densely overlapping canopies, ambiguous boundaries, and background clutter give rise to systematic counting errors that cannot be resolved by deeper or larger RGB networks alone [25,28]. A recent systematic review of CNN-based individual tree crown analysis explicitly concludes that the potential for multimodal data fusion has not been well-explored and that combining RGB images with additional spectral or structural information could be useful in resolving errors caused by overlapping tree crowns, shadows, and background noise [7]. These observations motivate the use of additional structural information for tree count estimation.

Airborne LiDAR provides a structural counterpart to optical imagery: the resulting point cloud and its raster derivatives, in particular the Canopy Height Model (CHM), capture the explicit vertical organization of forest scenes and are largely insensitive to illumination and shadow. CHM has long been used for individual tree detection and crown delineation through local-maxima search and watershed segmentation [29,30,31] and provides direct cues for canopy height, crown apex location and within-crown height variation that are difficult to recover from RGB imagery alone. Building on this complementarity, the fusion of RGB imagery with LiDAR-derived height information has been thoroughly investigated in remote sensing tasks. In urban scene understanding, RGB and Digital Surface Model (DSM) have been jointly exploited for land cover semantic segmentation [32,33] and building extraction [34,35]. In forestry and agriculture, optical and LiDAR data have been combined for urban and natural tree species classification [36,37,38], individual tree detection and species mapping [28], and crop-yield prediction at the parcel level [39]. These studies consistently show that height-aware fusion improves discrimination in tasks where appearance alone is ambiguous and have led to a rich set of fusion architectures spanning early/late concatenation, gated and attention-based fusion, and Transformer-based multimodal encoders. Closer to the present work, MTCDNet recently integrated UAV RGB imagery with CHM through Transformer-based fusion for tree crown detection [28]. To the best of our knowledge, however, the joint exploitation of RGB and CHM within a density map-based framework for tree counting in complex forest scenes remains essentially unexplored, despite the strong evidence that this modality combination is informative for forest-related tasks.

Bringing RGB and CHM together for density map-based tree counting is non-trivial, because the two modalities differ substantially in feature distribution, discriminative patterns and reliability, and these differences interact with the difficulties of forest scenes in three coupled ways. First, RGB and CHM are informative under different conditions: RGB cues are most useful where canopy boundaries are visually distinct, whereas CHM is more reliable in shadowed and low-texture regions but tends to merge adjacent crowns of similar height into a single apex [28,40]. A static fusion strategy, such as channel-wise concatenation or globally fixed weighting, cannot accommodate this spatially varying reliability. Second, simple unidirectional fusion, in which one modality is used only to enhance the other, ignores the bidirectional nature of the cross-modal evidence and is known to be sub-optimal in RGB-Depth segmentation and counting [41,42,43]. Third, the supervision signal in density map counting is inherently weak: each tree is represented by a point or a box derived density blob, providing little explicit guidance for the multimodal encoder to actually use both modalities, so that one branch may collapse onto the dominant modality during training. Together, these challenges motivate a tree counting framework in which RGB and CHM interact bidirectionally and adaptively, and in which the fused representation is explicitly regularized so that information from both modalities is preserved.

To address these challenges in a unified manner, we propose BCAR-Net (Bidirectional Cross-Attention with auxiliary Reconstruction Network), a broker-guided RGB-Depth framework tailored to density-map tree counting in complex forest scenes. Inspired by the broker-modality idea recently introduced for multimodal crowd counting [44] but specialized for RGB and CHM, BCAR-Net treats multimodal fusion as a structured interaction between appearance and geometry rather than as a passive combination of pre-computed features. The proposed framework contains four key components, the first three addressing the architectural challenges identified above and the fourth addressing the supervision design. (i) Broker generation via a bidirectional cross-attention U-Net. A bidirectional cross-attention U-Net (BCA-UNet) generates an intermediate broker RGB-D representation by computing symmetric RGB→Depth and Depth→RGB cross-attention, with a direction-aware gate adaptively weighting the two interaction paths. (ii) Tri-branch adaptive fusion. The original RGB image, depth map and broker representation are jointly encoded by three weight-sharing branches built on a shared VGG-ViT backbone and aggregated by a spatial fusion gate that emphasizes RGB cues in regions with clear canopy boundaries, depth cues in occluded or low-texture areas, and the broker representation in regions where a cross-modal summary is more informative. (iii) Multi-scale cross-attention reconstruction and two-stage training. A multi-scale cross-attention reconstruction decoder regularizes the fused latent feature through auxiliary RGB and depth reconstruction from multi-scale BCA-UNet encoder features, and a reconstruction-oriented two-stage training scheme replaces externally generated fused-image supervision [45] with self-reconstruction, providing a task-consistent initialization that avoids inheriting artifacts from external fusion procedures. Extensive experiments on the NEONTreeEvaluation benchmark and an additional UAV RGB-LiDAR test set demonstrate that BCAR-Net consistently outperforms representative single-modality baselines, direct RGB-D concatenation, a YOLO-based detection baseline and a transferred broker-modality multimodal baseline, with ablation studies confirming that each of the four designs contributes to the final performance.

The main contributions of this work are summarized as follows:

- We propose BCAR-Net, the first multimodal deep learning framework, to the best of our knowledge, that fuses RGB imagery and LiDAR-derived CHM for tree counting in complex forest scenes through a unified density map-based pipeline.
- We design a BCA-UNet for broker generation, which performs symmetric RGB↔Depth cross-attention and direction-aware gating, enabling deeper and more balanced cross-modal interaction than conventional unidirectional or static fusion.
- We introduce a tri-branch encoder with a spatial fusion gate, which adaptively assigns spatial importance to the RGB, depth and broker RGB–D branches, allowing the most reliable source of evidence to be emphasized under varying canopy densities and imaging conditions.
- We develop a multi-scale cross-attention reconstruction decoder together with a reconstruction-oriented two-stage training scheme, which replaces externally generated fused-image supervision with self-reconstruction of RGB and depth and yields a task-consistent optimization scheme for RGB-D tree counting.

## 2. Materials and Methods

### 2.1. Datasets and Preprocessing

**Figure 1:**
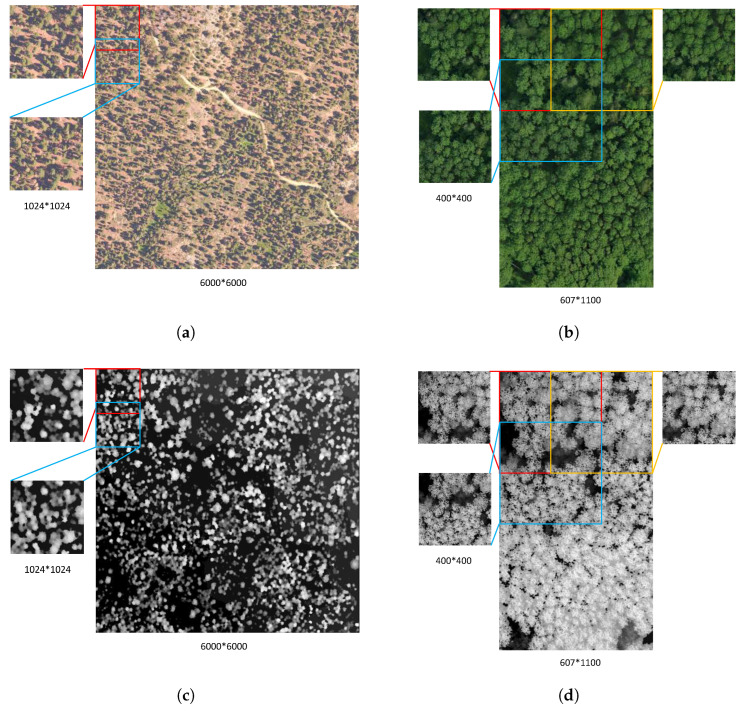
Representative examples of the multimodal dataset used in this study. Panels (**a**,**c**) are sampled from the NEON training set, while panels (**b**,**d**) are sampled from the public UAV RGB–LiDAR dataset. Panels (**a**,**b**) show RGB imagery, and panels (**c**,**d**) show the corresponding depth-related observations.

The multimodal data used in this study were derived from two sources: the NEONTreeEvaluation benchmark and a public UAV RGB–LiDAR dataset. The NEONTreeEvaluation benchmark provides co-registered airborne RGB imagery, LiDAR-derived products, hyperspectral observations, and crown annotations across multiple forest sites [6]. In this study, only RGB imagery and height-related structural information were used. The hyperspectral modality was not included in the experiments. The UAV samples were obtained from the public dataset of Dubrovin et al. [46,47], which provides UAV LiDAR point clouds, RGB orthophotos, and field-survey tree locations for dense mixed forests in Perm Krai, Russia. Representative RGB and depth-related observations from the two datasets are shown in Figure 1.

For both datasets, the depth input refers to a canopy-height-related structural representation rather than camera depth. CHM was used as the primary height representation because it directly describes the canopy height above ground. When both CHM and LiDAR-derived height information were available, valid pixels from the two sources were normalized to a common range and combined to form a unified single-channel depth image. When LiDAR-derived height information was unavailable or incomplete, the CHM map alone was used as the depth input. This preprocessing strategy provides a consistent RGB–Depth input format while preserving the physical meaning of the height modality. As illustrated in Figure 1, the RGB images provide appearance and texture information, while the depth-related observations describe the canopy-height structure and spatial variation. The spatial resolution of the input data was also recorded, because it affects crown visibility and the quality of height-structure cues.

The NEON samples were generated from the benchmark training and evaluation subsets separately. For training, aligned RGB–Depth tiles were cropped into 1024×1024 patches using a fixed sliding-window strategy. Overlap between neighboring patches was allowed only during training patch extraction to enrich the local canopy contexts seen by the model. Patches cropped from the same source tile were kept within the same data split, and no overlapping crop augmentation was applied to the test set. The NEON test set was constructed separately from the benchmark evaluation subset and was used only for quantitative evaluation. After removing invalid or incomplete samples, 937 training patches and 189 testing samples were retained. Training and testing data were therefore separated before patch generation, and the reported test results were obtained on samples that were not cropped from the same source tiles as the training patches.

The retained NEON samples cover multiple sites with different canopy structures and imaging conditions. Table 1 summarizes the site-level composition of the NEON samples after preprocessing. The training set contains samples from six NEON sites, whereas the test set includes samples from twenty NEON sites. The training subset is mainly composed of SJER, OSBS, TOOL, and TEAK samples, while the evaluation subset contains a broader set of sites with smaller numbers of samples per site.

For the UAV RGB–LiDAR dataset, the original data were collected from a dense mixed forest study area in Perm Krai, Russia. The dataset contains UAV LiDAR point clouds, RGB orthophotos, and field-survey tree locations. The field inventory contains 3600 trees distributed across 10 rectangular ground plots, each with a size of 100m×50m [46]. In this study, the UAV data were treated as a single-site dataset, because all available samples originated from the same study area. RGB images and depth-related structural data were aligned and cropped into 400×400 patches. After preprocessing and filtering, 129 UAV samples were retained, including 72 training samples and 57 testing samples.

Table 2 summarizes the final data construction protocol used in this study, including the native spatial resolution or point density of the input data and the approximate ground coverage of each patch. These resolution differences may affect the counting quality, since finer RGB resolution helps preserve crown texture and boundary details, whereas coarser height products may smooth local height discontinuities between neighboring crowns.

### 2.2. BCAR-Net

BCAR-Net formulates tree counting as an RGB-D multimodal density regression problem. Given a paired RGB image and depth map, the model estimates a density map whose spatial integral corresponds to the predicted number of trees in the observed region. This density-based formulation follows the general principle of density map regression in object counting, where spatially distributed supervision is preferred over direct scalar regression because it preserves the local counting structure and is more robust to overlap and clutter [13].

**Figure 2:**
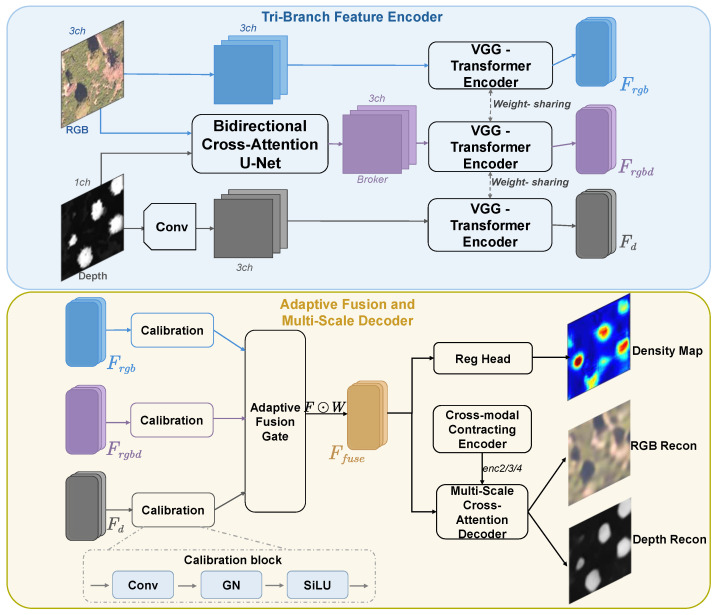
Illustration of the proposed BCAR-Net framework. By introducing an intermediate broker RGB–D representation, the original RGB and depth modalities are transformed into a tri-branch multimodal learning pipeline for adaptive fusion and density-map-based counting. An auxiliary reconstruction decoder regularizes the fused latent feature during training.

As shown in Figure 2, BCAR-Net consists of four key components that correspond directly to the following method sections and ablation studies: broker generation via a bidirectional cross-attention U-Net, tri-branch adaptive fusion, multi-scale cross-attention reconstruction with two-stage training, and bounding-box-guided density supervision. The original RGB image and depth map are first used to generate an intermediate broker RGB–D representation. The original modalities and the broker representation are then encoded by three weight-sharing branches and adaptively fused for density regression. During training, a reconstruction decoder regularizes the fused latent feature by reconstructing RGB and depth observations.

Let $I_{\mathrm{rgb}}\in R^{3\times H\times W}$ denote an RGB image and $I_{d}\in R^{1\times H\times W}$ denote the corresponding single-channel depth map. The goal is to learn a mapping

(1) $$\;\hat{D}=F\left(I_{\mathrm{rgb}},I_{d};\theta \right),$$

where F(·) denotes the BCAR-Net counting network with parameters θ, and $\hat{D}\in R^{1\times h\times w}$ is the predicted density map. The final tree count is obtained by summing the density values over the spatial domain:

(2) $$\hat{C}=\sum_{x=1}^{w}\sum_{y=1}^{h}\hat{D}\left(x,y\right).$$

This density-based formulation is adopted instead of direct scalar regression because it preserves spatial information regarding the distribution of trees and provides stronger supervision in dense and heterogeneous forest scenes [13]. Since the tree annotations are provided as bounding boxes, each box is converted into a density target for density-map regression. The bounding boxes are not used for direct object detection; instead, they are used only to construct the supervision map for counting. Let $\Omega_{j}$ denote the spatial region covered by the *j*-th annotated box, and let $a_{j}$ denote its mass contribution. The target density map is defined as

(3) $$\;D_{\mathrm{box}}^{*}\left(x,y\right)=\sum_{j=1}^{M}\rho_{j}\left(x,y\right),$$

where *M* is the number of valid annotated boxes in the image, and $\rho_{j}\left(x,y\right)$ denotes the density distribution generated from the *j*-th box. In principle, $\rho_{j}\left(x,y\right)$ can be constructed either as a uniform distribution within the box region or as a Gaussian distribution centered at the box location and normalized within the corresponding box region. In this study, the Gaussian box-density target is used in all main experiments, while the uniform box-density target is considered only as an ablation variant. In both cases, the total mass assigned to each valid annotation is preserved, so that the integral of the target density map corresponds to the ground-truth tree count. This target construction allows the model to remain within the density-regression framework while using the available bounding-box annotations. Although rectangular boxes may include some background pixels for irregular tree crowns, they provide a practical way to generate spatially distributed supervision for training the counting network.

#### 2.2.1. Broker Generation via a Bidirectional Cross-Attention U-Net

**Figure 3:**
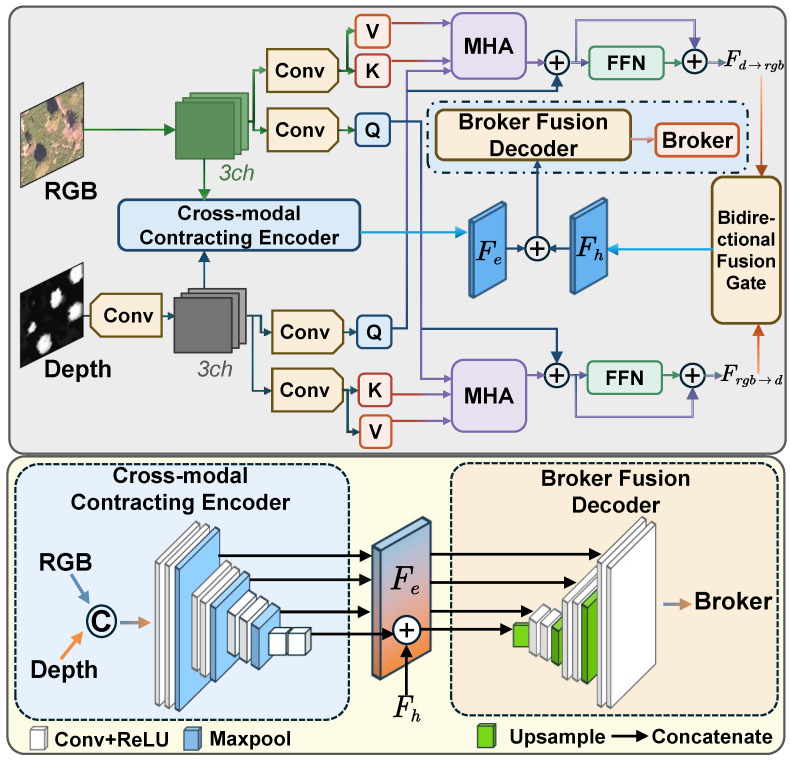
Architecture of BCA-UNet. Single-channel depth is first adapted to a three-channel representation, after which RGB→Depth and Depth→RGB cross-attention are computed and fused by a direction-aware gate. The resulting cross-modal feature is injected into the deepest U-Net stage to produce the intermediate broker RGB-D representation.

Direct fusion of RGB and depth modalities is often suboptimal because the two modalities differ substantially in feature distribution, discriminative patterns, and noise characteristics. To alleviate this issue, the BCA-UNet is designed to generate an intermediate broker RGB-D representation from the paired RGB image and depth map. The detailed architecture is shown in Figure 3. The overall U-shaped encoder–decoder structure follows U-Net [48], while the cross-modal interaction module is implemented using Transformer-style cross-attention [49]. The single-channel depth map is first projected to a three-channel representation before interacting with the RGB stream.

Let $X_{\mathrm{rgb}}$ and $X_{d}$ denote the shallow features extracted from the RGB and adapted depth inputs, respectively. Two complementary cross-modal interaction paths are constructed and then fused by a direction-aware gate:

(4) $$\begin{array}{cc} Att_{\mathrm{rgb}\to d} & =\mathrm{CA}\left(X_{\mathrm{rgb}},X_{d}\right),\;Att_{d\to \mathrm{rgb}}=\mathrm{CA}\left(X_{d},X_{\mathrm{rgb}}\right), \end{array}$$

(5) $$\begin{array}{cc} \left[\alpha_{1},\alpha_{2}\right] & =\mathrm{Softmax}\left(\psi (\left[Att_{\mathrm{rgb}\to d};Att_{d\to \mathrm{rgb}}\right])\right), \end{array}$$

(6) $$\begin{array}{cc} Att_{\mathrm{cross}} & =\alpha_{1}⊙Att_{\mathrm{rgb}\to d}+\alpha_{2}⊙Att_{d\to \mathrm{rgb}}, \end{array}$$

where CA(·,·) denotes a cross-attention block, in which one modality provides the query representation, the other provides the key/value representation, and ψ(·) denotes a lightweight 1×1 convolutional gating network.

The cross-modal enhancement is injected into the U-Net bottleneck, and the intermediate broker RGB–D representation is generated by the decoder:

(7) $$I_{\mathrm{rgbd}}=G_{\mathrm{unet}}\left(I_{\mathrm{rgb}},I_{d};Att_{\mathrm{cross}}\right),$$

where $G_{\mathrm{unet}}\left(\cdot \right)$ denotes BCA-UNet. This output is not used as a replacement for the original modalities. Instead, it acts as a broker representation that bridges appearance and geometry for downstream tri-branch fusion.

#### 2.2.2. Tri-Branch Adaptive Fusion

After generating the broker RGB–D representation $I_{\mathrm{rgbd}}$, the original RGB image, the depth map, and the broker representation are jointly encoded by three weight-sharing branches built upon a shared VGG-ViT backbone [50,51]. The depth map is first expanded to a three-channel representation, denoted as ${\tilde{I}}_{d}$, for compatibility with the shared backbone. The branch-wise features are extracted as

(8) $$\begin{array}{cc} F_{\mathrm{rgb}} & =C_{\mathrm{rgb}}\;\left(\Psi (\Phi \left(I_{\mathrm{rgb}}\right))\right), \end{array}$$

(9) $$\begin{array}{cc} F_{d} & =C_{d}\;\left(\Psi (\Phi \left({\tilde{I}}_{d}\right))\right), \end{array}$$

(10) $$\begin{array}{cc} F_{\mathrm{rgbd}} & =C_{\mathrm{rgbd}}\;\left(\Psi (\Phi \left(I_{\mathrm{rgbd}}\right))\right), \end{array}$$

where Φ(·) denotes the shared VGG-style convolutional feature extractor, Ψ(·) denotes the lightweight Vision Transformer encoder, and C(·) denotes the branch-specific calibration block composed of 1×1 convolution, Group Normalization, and SiLU activation.

The three branch features are adaptively aggregated by a spatial fusion gate:

(11) $$\begin{array}{cc} \left[w_{\mathrm{rgb}},w_{d},w_{\mathrm{rgbd}}\right] & =\mathrm{Softmax}\;\left(\gamma (\left[F_{\mathrm{rgb}},F_{d},F_{\mathrm{rgbd}}\right])\right), \end{array}$$

(12) $$\begin{array}{cc} F_{\mathrm{fuse}} & =w_{\mathrm{rgb}}⊙F_{\mathrm{rgb}}+w_{d}⊙F_{d}+w_{\mathrm{rgbd}}⊙F_{\mathrm{rgbd}}, \end{array}$$

where γ(·) is implemented by lightweight 1×1 convolutions. This spatially varying gating allows the model to emphasize RGB cues, depth cues, or the broker representation according to local modality reliability.

The fused feature map is finally fed to a three-layer convolutional regression head to predict the one-channel density map:

(13) $$\hat{D}=R\left(F_{\mathrm{fuse}}\right).$$

#### 2.2.3. Multi-Scale Cross-Attention Reconstruction

**Figure 4:**
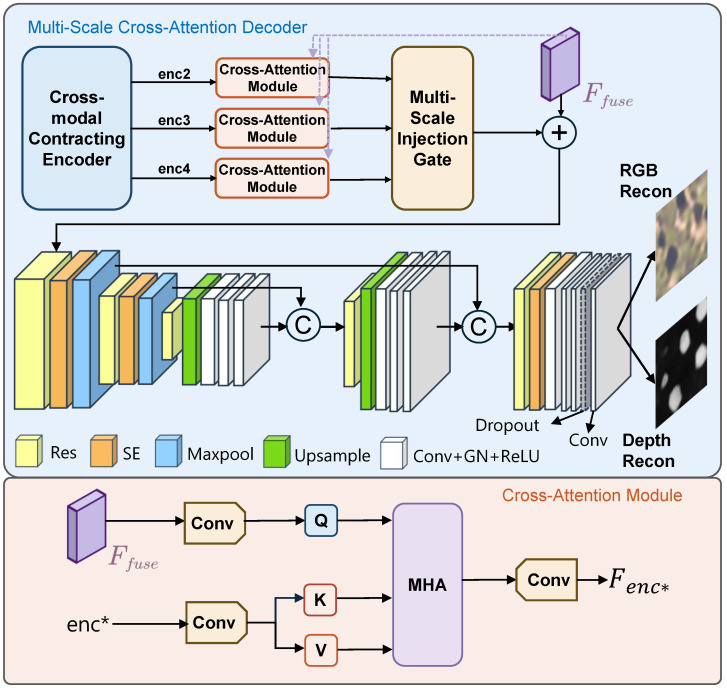
Architecture of the multi-scale cross-attention reconstruction decoder. The fused latent feature queries intermediate BCA-UNet encoder features through 2D cross-attention. The injected features are weighted by independent sigmoid gates and decoded by a two-level U-Net-like pathway to reconstruct RGB and depth observations.

To regularize the fused latent feature, a multi-scale cross-attention reconstruction decoder reuses intermediate BCA-UNet encoder features to provide auxiliary RGB and depth reconstruction supervision. The detailed architecture is illustrated in Figure 4. Instead of using externally generated fused images as supervision targets, the fused latent feature is decoded into the original modalities.

Let $E=\{E_{2},E_{3},E_{4}\}$ denote the selected intermediate encoder features from BCA-UNet. For each scale e∈{2,3,4}, the fused feature provides the query representation, while the encoder feature provides the key and value representations:

(14) $$\begin{array}{cc} Q_{e} & =P_{q}^{\left(e\right)}\left(F_{\mathrm{fuse}}\right),\;K_{e}=P_{k}^{\left(e\right)}\left(E_{e}\right),\;V_{e}=P_{v}^{\left(e\right)}\left(E_{e}\right), \end{array}$$

(15) $$\begin{array}{cc} J_{e} & =\mathrm{Attention}\left(Q_{e},K_{e},V_{e}\right)=\mathrm{Softmax}\;\left(\frac{Q_{e}K_{e}^{⊤}}{\sqrt{d}}\right)V_{e}, \end{array}$$

where $P_{q}^{\left(e\right)}\left(\cdot \right)$, $P_{k}^{\left(e\right)}\left(\cdot \right)$, and $P_{v}^{\left(e\right)}\left(\cdot \right)$ denote learnable 1×1 projection layers, and *d* is the feature dimension.

The reconstruction decoder uses independent sigmoid gates, so that multi-scale features can contribute simultaneously rather than competitively:

(16) $$\begin{array}{cc} \left[g_{2},g_{3},g_{4}\right] & =\sigma (\Gamma \left(F_{\mathrm{fuse}}\right)), \end{array}$$

(17) $$\begin{array}{cc} F_{0} & =F_{\mathrm{fuse}}+g_{2}⊙J_{2}+g_{3}⊙J_{3}+g_{4}⊙J_{4}, \end{array}$$

where Γ(·) is a lightweight gating network, and σ(·) denotes the sigmoid function.

The injected feature $F_{0}$ is then passed through a two-level U-Net-like reconstruction pathway. As illustrated in Figure 4, the reconstruction pathway first applies residual feature extraction and channel recalibration to refine the injected feature. Specifically, residual convolution (Res) blocks are used to enhance local feature representation, while squeeze-and-excitation (SE) blocks recalibrate channel responses through global pooling and learned channel weights. This design helps the decoder emphasize informative channels related to appearance and structural recovery.

The deepest feature in the reconstruction pathway is then progressively upsampled and fused with the corresponding skip features. Each upsampling block consists of bilinear upsampling followed by convolution, normalization, and nonlinear activation, and the upsampled features are concatenated with skip features from the reconstruction pathway. Through this encoder–decoder structure, the decoder combines deep semantic information with structural details preserved in shallow and intermediate features.

Finally, the shared decoder feature is mapped to RGB and depth reconstructions through two lightweight output heads. These heads generate ${\hat{I}}_{\mathrm{rgb}}$ and ${\hat{I}}_{d}$, respectively. By reconstructing both RGB and depth observations from the same fused feature, the auxiliary reconstruction branch serves as a training-time regularizer that encourages the fused representation to encode task-relevant complementary cues from both modalities.

#### 2.2.4. Two-Stage Training Strategy

BCAR-Net is optimized using a two-stage training strategy. This strategy follows the high-level staged learning idea of broker modality training but replaces externally generated fused-image supervision with self-reconstruction. In the first stage, the optimization is driven mainly by RGB and depth reconstruction, while a weak counting regularizer is retained to keep the learned fused representation compatible with the downstream task. In the second stage, the whole network is jointly optimized for tree counting, while the auxiliary objectives are gradually weakened:

(18) $$\begin{array}{cc} L_{\mathrm{stage}1} & =\lambda_{\mathrm{rgb}}L_{\mathrm{rgb}}+\lambda_{d}L_{d}+\lambda_{\mathrm{con}}L_{\mathrm{con}}+\lambda_{\mathrm{cf}}L_{\mathrm{count}}, \end{array}$$

(19) $$\begin{array}{cc} L_{\mathrm{stage}2}\left(e\right) & =\lambda_{\mathrm{count}}L_{\mathrm{count}}+s\left(e\right)\left(\lambda_{\mathrm{rgb}}L_{\mathrm{rgb}}+\lambda_{d}L_{d}+\lambda_{\mathrm{con}}L_{\mathrm{con}}\right), \end{array}$$

where $\lambda_{\mathrm{cf}}$ is a small coefficient controlling the weak counting regularization, and s(e) is an epoch-dependent decay factor.

#### 2.2.5. Loss Functions

The overall training objective combines counting supervision, auxiliary reconstruction, and weak feature consistency regularization. The main counting objective consists of a densitymap loss derived from the bounding box annotations and an image-level count consistency term:

(20) $$L_{\mathrm{count}}=\lambda_{\mathrm{map}}{∥\hat{D}-D_{\mathrm{box}}^{*}∥}_{1}+\lambda_{\mathrm{cnt}}\left|\hat{C}-C^{*}\right|,$$

where $\hat{D}$ is the predicted density map, $D_{\mathrm{box}}^{*}$ is the density supervision map generated from the retained bounding boxes, $\hat{C}=\sum_{x,y}\hat{D}\left(x,y\right)$ is the predicted count, and $C^{*}$ is the ground-truth count derived from the retained box annotations.

The auxiliary reconstruction objective contains RGB reconstruction and depth reconstruction losses. The RGB reconstruction loss is defined as

(21) $$L_{\mathrm{rgb}}={∥{\hat{I}}_{\mathrm{rgb}}-I_{\mathrm{rgb}}∥}_{2}^{2},$$

where ${\hat{I}}_{\mathrm{rgb}}$ and $I_{\mathrm{rgb}}$ denote the reconstructed and input RGB images, respectively. The depth reconstruction loss combines pixel-wise fidelity, structural similarity (ssim), and gradient consistency (grad):

(22) $$L_{d}=\left(1-\beta_{\mathrm{ssim}}\right){∥{\hat{I}}_{d}-I_{d}∥}_{1}+\beta_{\mathrm{ssim}}L_{\mathrm{ssim}}+\beta_{\mathrm{grad}}L_{\mathrm{grad}},$$

where ${\hat{I}}_{d}$ and $I_{d}$ denote the reconstructed and input depth maps, respectively. $L_{\mathrm{ssim}}$ denotes the structural similarity loss based on SSIM [52], and $L_{\mathrm{grad}}$ denotes the $L_{1}$ difference between the spatial gradients of the reconstructed and input depth maps.

Although RGB imagery and CHM describe different physical properties, both modalities contain counting-related responses around tree crowns. Therefore, the feature consistency term is not imposed on the raw RGB and depth inputs. Instead, it is applied only to high-level task features after modality-specific encoding and channel calibration. The feature consistency loss is defined as

(23) $$L_{\mathrm{con}}={∥F_{\mathrm{rgb}}-F_{d}∥}_{1}+{∥F_{\mathrm{fuse}}-\frac{F_{\mathrm{rgb}}+F_{d}}{2}∥}_{1}.$$

This term is used as a weak task-oriented regularizer rather than as a constraint that forces the two modalities to become identical. It encourages RGB and depth features to share counting-related responses, while modality-specific information is preserved by the separate RGB, depth, and broker branches as well as the adaptive fusion gate. In all experiments, a small weight $\lambda_{\mathrm{con}}=0.02$ was used for this term.

### 2.3. Model Complexity and Regularization Considerations

BCAR-Net is designed as a modular architecture rather than as an undirected increase in model capacity. The main components map to three functional objectives: cross-modal interaction, global–local feature encoding, and auxiliary reconstruction regularization. The BCA-UNet module constructs an intermediate broker representation that explicitly exchanges information between RGB texture cues and height-related structural cues. The VGG–ViT encoder combines convolutional local feature extraction with Transformer-based long-range context modeling. The reconstruction branch is used only during training to regularize the fused latent representation and is removed at inference time.

Several implementation choices help mitigate the risk of overfitting on relatively small tree-counting datasets. First, the two-stage training strategy separates reconstruction-oriented representation learning from counting-oriented optimization, preventing the auxiliary reconstruction loss from dominating the final density map regression objective. Second, the reconstruction-related losses are gradually down-weighted in the second training stage. Third, the auxiliary reconstruction decoder adds no inference time computation because it is used only during training. Finally, data augmentation and validation-based model selection were applied during training.

The computational complexity of BCAR-Net and representative comparison models is summarized in Table 3. The number of parameters and floating-point operations (FLOPs) were measured using one 384×384 input sample with batch size 1. For BCAR-Net, the full training model contains 74 M parameters, while the inference-stage model contains 40 M parameters because the auxiliary RGB/Depth reconstruction decoders are removed during inference. Therefore, BM and BCAR-Net have the same inference-stage parameter count and FLOPs, although BCAR-Net contains additional training-time reconstruction parameters.

## 3. Results and Discussion

### 3.1. Implementation Details

BCAR-Net is implemented in PyTorch 1.10.2 and trained on a single NVIDIA RTX 5090 GPU with 32 GB of memory. Training uses the Adam optimizer with an initial learning rate of $1\times 10^{-5}$ and a weight decay of $1\times 10^{-4}$. The learning rate is decayed by a factor of 0.5 every 100 epochs starting from epoch 200. For bounding-box-guided density supervision, the Gaussian box-density target was used in all main experiments. The batch size is set to 8, and the model is trained for 400 epochs in total, including 50 epochs for the reconstruction-oriented first stage and 350 epochs for the counting-oriented second stage. The input crop size used during training is 384×384. The two-stage training scheme described in Section 2.2.4 was followed throughout. During the second stage, the auxiliary reconstruction loss weight was controlled by s(e): it was set to 1.0 before epoch 300, linearly reduced to 0.1 from epoch 300 to epoch 400, and then kept at 0.1 for the remaining epochs.

For the loss configuration, the main counting loss weight is set to $\lambda_{\mathrm{count}}=1$. In the counting objective, the density map loss and count consistency loss are weighted by $\lambda_{\mathrm{map}}=2$ and $\lambda_{\mathrm{cnt}}=1$, respectively. In the auxiliary objectives, the RGB reconstruction, depth reconstruction, and feature consistency terms are weighted by $\lambda_{\mathrm{rgb}}=0.2$, $\lambda_{d}=0.3$, and $\lambda_{\mathrm{con}}=0.02$, respectively. During the first training stage, a weak counting regularization term is retained with $\lambda_{\mathrm{cf}}=0.1$. For depth reconstruction, the SSIM and gradient consistency terms are weighted by $\beta_{\mathrm{ssim}}=0.5$ and $\beta_{\mathrm{grad}}=0.05$.

### 3.2. Evaluation Metrics and Experimental Settings

BCAR-Net is evaluated using three widely adopted regression metrics, namely the Mean Absolute Error (MAE), Root Mean Squared Error (RMSE), and the coefficient of determination ($R^{2}$). MAE measures the average absolute deviation between the predicted and ground-truth tree counts, while RMSE places more emphasis on larger prediction errors. In contrast, $R^{2}$ reflects the overall goodness of fit between predicted and observed counts. For quantitative evaluation, we used the NEONTreeEvaluation test subset [6].

Let ${\hat{C}}_{i}$ and $C_{i}$ denote the predicted and ground-truth tree counts for the *i*-th test sample, respectively, and let *N* be the total number of test samples. The three evaluation metrics are defined as

(24) $$\mathrm{MAE}=\frac{1}{N}\sum_{i=1}^{N}\left|{\hat{C}}_{i}-C_{i}\right|,$$

(25) $$\mathrm{RMSE}=\sqrt{\frac{1}{N}\sum_{i=1}^{N}{\left({\hat{C}}_{i}-C_{i}\right)}^{2}},$$

(26) $$R^{2}=1-\frac{\sum_{i=1}^{N}{\left(C_{i}-{\hat{C}}_{i}\right)}^{2}}{\sum_{i=1}^{N}{\left(C_{i}-\bar{C}\right)}^{2}},$$

where $\bar{C}$ denotes the mean of the ground-truth counts over the test set.

### 3.3. Comparative Experiments

To assess the overall effectiveness of BCAR-Net, we conduct two test experiments under different evaluation settings. In the first experiment, the model is trained on the NEON training set and evaluated on the NEON test set. In the second experiment, the model trained on the NEON training set is further fine-tuned on the UAV training set and then evaluated on the UAV test set. The compared methods cover several different design paradigms, including generic CNN-based backbones, stronger counting-oriented baselines, and the broker modality baseline BM. Specifically, SENet50 is based on channel-wise feature recalibration [53], ResNet50 is a standard residual CNN backbone [54], STEERER is designed to address scale variation in counting and localization [55], APGCC improves point-based counting by stabilizing proposal–target matching with auxiliary point guidance [56], P2RLoss introduces point-to-region supervision for point-based crowd counting [57], and BM is the most relevant multimodal baseline [44]. This comparison is intended to examine not only whether multimodal learning is beneficial but also whether the proposed RGB–D formulation is more suitable for tree counting than both unimodal counters and the original broker-style design.

A notable difference between BCAR-Net and several compared counting baselines lies in the supervision form. Some existing methods are primarily designed for point-style supervision [56,57], where each object is represented by a single annotated location. Such supervision is efficient and well suited to standard counting settings, but it provides limited information about the object extent. By contrast, BCAR-Net adopts bounding-box-guided density supervision, which preserves the density-regression formulation while introducing additional spatial cues about the canopy extent.

Experimental results on NEONTreeEvaluation dataset. The overall quantitative comparison on the NEON test set is summarized in Table 4. BCAR-Net achieves the best performance, reaching an MAE of 7.92, an RMSE of 14.20, and an $R^{2}$ of 0.8684. These results indicate that the proposed framework yields not only a lower counting error but also a better regression fit than the competing methods.

Within the RGB-only group, SENet50 and ResNet50 provide standard convolutional feature extraction, but their performance remains limited in cluttered forest scenes, suggesting that generic backbone enhancement alone is insufficient for reliable tree counting under canopy overlap and heterogeneous backgrounds. In addition to density-regression baselines, a YOLO-based detector was included as a detection-based counting baseline using RGB input, and its predicted count was obtained by counting the detected crown boxes after post-processing. As reported in Table 4, YOLO outperforms SENet50, ResNet50, and STEERER under RGB-only input, confirming that the original bounding-box annotations are useful for direct crown detection. Its counting error nevertheless remains higher than that of BCAR-Net, indicating that multimodal density regression keeps a clear advantage for count estimation when RGB texture and height-related structural information are jointly used. In the depth-only group, STEERER gives the strongest unimodal performance, indicating that geometric cues are particularly informative for the NEON scenes. Even so, BCAR-Net further reduces the MAE from 11.21 to 7.92 and RMSE from 22.67 to 14.20, showing that multimodal fusion still provides clear gains beyond depth alone.

The most important multimodal comparison is with BM in the RGB+Depth category. As shown in Table 4, BM also benefits from multimodal input, but its performance remains clearly inferior to that of BCAR-Net. This gap suggests that directly transferring the original broker modality formulation to RGB–D tree counting is not sufficient. In our case, the gain appears to come from several coordinated modifications, including bidirectional cross-attention in the broker generation stage, explicit tri-branch encoding of RGB, Depth, and broker RGB–D features, adaptive spatial gating during branch fusion, and a reconstruction-oriented staged training strategy.

**Figure 5:**
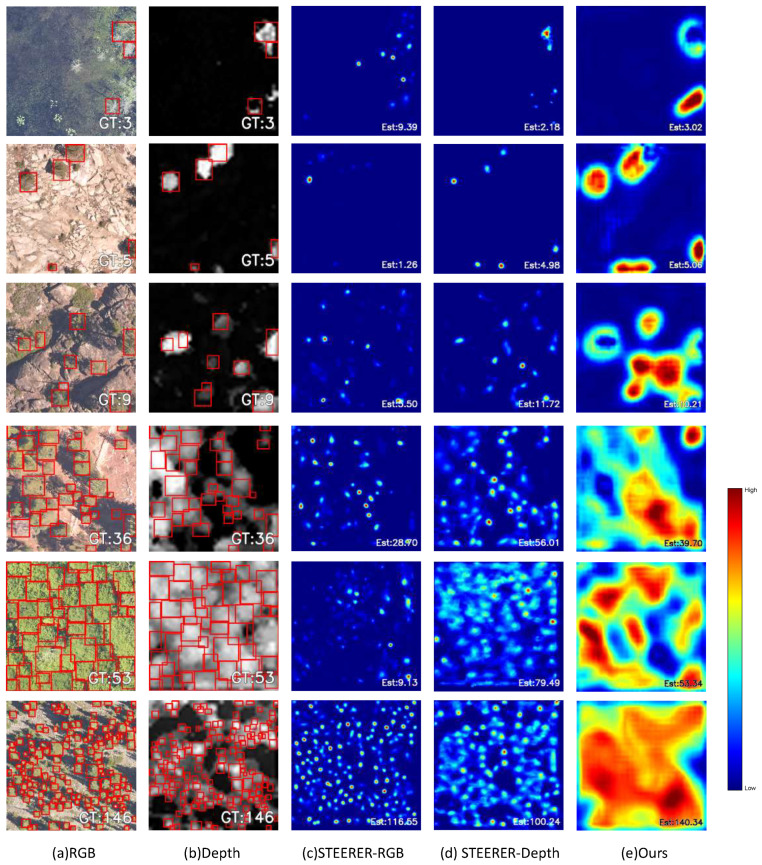
Visual comparison of predicted density maps and tree-counting results on representative forest plots from the NEONTreeEvaluation test set. The samples are arranged from sparse to dense forest stands from top to bottom, illustrating the performance of different methods under varying canopy closure conditions.

Furthermore, qualitative results on the NEON test set are presented in Figure 5 to further illustrate the behavior of BCAR-Net under different forest conditions. The selected examples span a range of canopy densities and scene complexities, providing an intuitive complement to the quantitative results. Compared with the single-modality baselines STEERER-RGB and STEERER-Depth, BCAR-Net produces density maps whose spatial distribution is better aligned with the true canopy layout, and its predicted counts stay closer to the ground truth in both sparse and dense scenes.

Experimental results on UAV dataset. To further examine the behavior of BCAR-Net under a different acquisition setting, the model trained on the NEON training set is further fine-tuned on the UAV training set and then evaluated on the UAV test set. The quantitative comparison is reported in Table 5. To account for the small size of the UAV dataset, the compared methods were independently repeated multiple times, and the results are reported as the mean ± standard deviation. BCAR-Net achieves the best average performance, with an MAE of 11.56 ± 0.32, an RMSE of 14.70 ± 0.33, and an $R^{2}$ of 0.7360 ± 0.0127. Compared with P2RLoss-Depth, BCAR-Net obtains a lower average MAE and RMSE and a higher average $R^{2}$, although the improvement is relatively modest and should be interpreted cautiously given the small test set.

A notable trend on the UAV test set is that the depth-based baselines are stronger than the RGB-only baseline. STEERER-RGB shows large variability and a negative average $R^{2}$, whereas STEERER-Depth achieves lower average counting errors and a higher average $R^{2}$. P2RLoss-Depth further improves over STEERER-Depth, indicating that structural cues from the depth-related modality are especially informative under the UAV setting. BCAR-Net achieves the best average results among the compared methods, suggesting that multimodal fusion can further refine geometric cues with complementary appearance information after adaptation to the UAV data. However, given the limited number of UAV samples and the modest margin over P2RLoss-Depth, this experiment should be interpreted as a small-scale supplementary evaluation rather than as conclusive evidence of broad cross-platform generalization.

**Figure 6:**
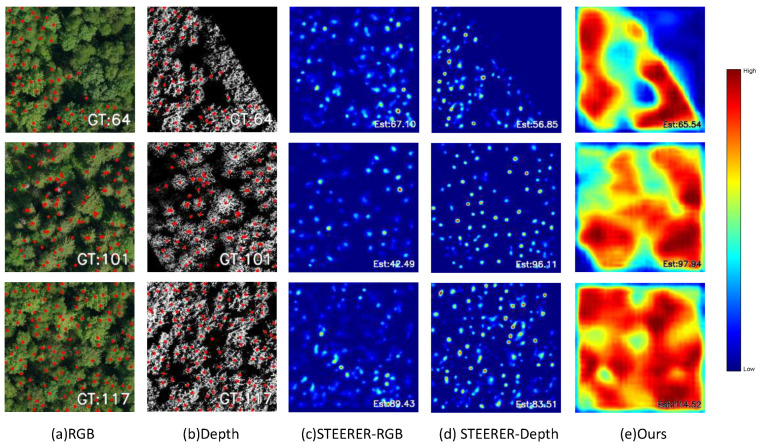
Qualitative comparison of representative tree-counting results on the UAV test set. From left to right are the RGB image, depth map, density maps predicted by STEERER-RGB, STEERER-Depth, and the proposed method. Ground-truth tree locations are marked in red. The estimated count (Est.) is obtained by integrating the original unnormalized density map. For visualization, each density map is independently normalized and rendered using the same colormap.

The UAV qualitative results are shown in Figure 6. Compared with the RGB-only and depth-only baselines, BCAR-Net produces more coherent density responses over tree-populated regions and gives predicted counts that are generally closer to the ground truth. These visual examples are used as qualitative illustrations, while the quantitative comparison is based on the full UAV test set in Table 5.

Overall, the two test experiments support two observations. First, multimodal learning is more effective than relying on a single modality alone in the evaluated settings, although the relative contribution of RGB and depth differs across datasets. Second, after adaptation to the UAV training data, BCAR-Net still benefits from the coordinated design of broker generation, tri-branch adaptive fusion, reconstruction-oriented training, and bounding-box-guided density supervision. Nevertheless, because the UAV dataset contains only a limited number of training and testing patches, the UAV results should be regarded as supplementary evidence of applicability under a different acquisition condition rather than a definitive demonstration of broad operational generalization.

### 3.4. Systematic Error Analysis

The visual examples in Figure 5 and Figure 6 are used only as qualitative illustrations. To provide a more systematic view of model behavior, we further grouped the NEONTreeEvaluation test samples by tree density, crown size, canopy closure, and site identity. Tree density was defined as the ground-truth tree count of each patch. Crown size was estimated by the average normalized area of annotated crown bounding boxes in each patch. Canopy closure was approximated by the proportion of valid depth pixels whose normalized height response was larger than 0.5. Therefore, this canopy-closure value should be interpreted as a height-map-based proxy rather than a field-measured canopy-closure variable. For tree density, crown size, and canopy closure, the samples were divided into low, medium, and high groups using tertiles computed on the test set.

For each group, the MAE, RMSE, $R^{2}$, and mean bias were reported. The bias is defined as

(27) $$\mathrm{Bias}=\frac{1}{N}\sum_{i=1}^{N}\left({\hat{C}}_{i}-C_{i}\right),$$

where ${\hat{C}}_{i}$ and $C_{i}$ denote the predicted and ground-truth counts of sample *i*, respectively. Positive bias indicates over-counting, while negative bias indicates under-counting.

Several patterns can be observed from Table 6. Among the analyzed factors, tree density shows one of the clearest error trends. The counting error increases substantially from low- and medium-density patches to high-density patches, where the MAE and RMSE reach 17.19 and 23.15, respectively. The negative bias of −9.21 further indicates that errors in dense patches are dominated by under-counting. This result suggests that dense crowns and overlapping canopy structures remain a primary failure mode for density map regression in complex forest scenes.

Crown size provides a related explanation for this behavior. Samples dominated by smaller annotated crowns produce larger errors, possibly because small crowns have weaker spatial support and are more easily confused with background texture or neighboring crowns in aerial imagery. In contrast, patches with larger average crown boxes have lower absolute errors. The negative $R^{2}$ in the large-crown group should be interpreted cautiously, since the within-group variation of ground-truth counts is relatively limited and can make $R^{2}$ unstable.

Canopy closure shows a similarly important error pattern. Low- and medium-closure patches have relatively small errors, whereas high-closure patches show a marked increase in the MAE and RMSE, reaching 17.17 and 23.50, respectively. The negative bias of −6.17 indicates that closed-canopy conditions also mainly lead to under-counting. This is likely because closed-canopy areas often contain visually connected neighboring crowns, while height discontinuities in the depth map may become less distinct. These conditions make individual crown contributions harder to separate and identify high-canopy-closure patches as another key limitation of the current model.

Site-level results further indicate that the performance is affected by local scene characteristics. The model performs well on SJER and TEAK, while larger errors are observed on NIWO and the merged group of other sites. Such variation may be related to differences in forest structure, crown morphology, illumination conditions, and depth-map quality. Overall, this grouped analysis provides a more systematic complement to the selected qualitative examples and clarifies where the current model is more likely to fail.

### 3.5. Ablation Studies

The ablation study was designed to evaluate whether each architectural component contributes to the final counting performance. Starting from an RGB-only baseline, depth information, broker-based cross-modal interaction, adaptive fusion, auxiliary reconstruction, and the two-stage training strategy were added progressively. This incremental evaluation helps distinguish the effect of the additional height modality from the effect of the proposed fusion and reconstruction mechanisms. To better understand where the performance gains come from, we analyze BCAR-Net according to the four main components introduced in the Introduction and Methods:

Broker generation via a bidirectional cross-attention U-Net. We first examine whether the gain mainly comes from multimodal input itself or from the intermediate broker RGB–D representation. The corresponding results are listed in Table 7. Both single-modality settings perform worse than multimodal learning on the NEONTreeEvaluation test set. Depth alone achieves a lower MAE than RGB alone, while RGB obtains a slightly higher $R^{2}$, suggesting that the two modalities provide different but complementary cues. Directly concatenating RGB and depth already produces a clear improvement over either single modality, confirming the importance of incorporating canopy-height information. Compared with simple RGB–D concatenation, the proposed RGB–D design further reduces the average MAE from 8.87 to 8.18 and the average RMSE from 15.79 to 14.52, while increasing the average $R^{2}$ from 0.8289 to 0.8624. Given the standard deviations, this improvement should be read as a consistent but relatively modest gain over direct concatenation. The largest gain therefore comes from introducing the height modality, while the broker generation design further refines RGB–Depth interaction by organizing the two modalities into an intermediate cross-modal representation.

We further evaluate the role of bidirectional cross-attention inside BCA-UNet. As shown in Table 8, removing the U-Net cross-attention or replacing it with a single-direction attention variant degrades the performance compared with the full model. Removing cross-attention increases the MAE from 7.92 to 8.38, while the single-direction variant further increases the MAE to 8.72. These results indicate that cross-modal interaction inside BCA-UNet is necessary and that the RGB–Depth relationship should not be modeled as a one-way information transfer. The bidirectional design enables more balanced interaction between appearance and geometric cues during broker generation.

Tri-branch adaptive fusion. We next evaluate the tri-branch adaptive fusion design, including the shared VGG–ViT branch encoder, the broker branch, and the spatial fusion gate. Since the three branches are built upon a shared VGG–ViT backbone, we first investigate how much Transformer capacity is needed. The results in Table 9 show that introducing a lightweight Transformer encoder is beneficial, but increasing its depth beyond one layer becomes counterproductive. Compared with the CNN-only setting, one Transformer layer reduces the MAE from 9.11 to 7.92 and RMSE from 16.25 to 14.20. This indicates that moderate contextual modeling helps tree counting, while deeper Transformer stacks may introduce unnecessary optimization difficulty for the current dataset scale.

The structural contribution of tri-branch adaptive fusion is further analyzed in Table 10. Disabling the spatial fusion gate causes a clear performance drop, increasing the MAE to 9.21 and the RMSE to 16.31. This indicates that fixed or less adaptive fusion is insufficient for handling spatially varying modality reliability. Removing the broker RGB–D branch also degrades performance, with the MAE increasing to 8.23 and the RMSE increasing to 14.40. Although this degradation is smaller than that caused by removing the fusion gate, it still shows that the broker representation contributes useful cross-modal information to the tri-branch fusion framework.

Multi-scale cross-attention reconstruction and two-stage training. We then examine the reconstruction module and the staged optimization strategy. First, we evaluate the cross-attention design in the reconstruction decoder. The results in Table 11 show that removing decoder-side cross-attention increases the MAE from 7.92 to 9.28 and the RMSE from 14.20 to 16.12. This clear degradation suggests that the reconstruction branch benefits from selectively injecting intermediate BCA-UNet encoder features, rather than decoding only from the fused latent feature.

The influence of auxiliary reconstruction supervision is summarized in Table 12. Removing all reconstruction losses degrades the model, confirming that reconstruction acts as an effective regularizer for multimodal fusion learning. Among the two reconstruction branches, removing depth reconstruction causes a larger performance drop than removing RGB reconstruction, which suggests that geometry-aware reconstruction is especially important for stabilizing the fused representation in tree counting. At the same time, removing RGB reconstruction is also harmful, indicating that appearance recovery still contributes to the learned representation. The feature consistency term contributes a smaller but still measurable amount. Removing it raises the MAE from 7.92 to 8.20, raises the RMSE from 14.20 to 15.07, and lowers $R^{2}$ from 0.8684 to 0.8517, showing that weak task-level consistency between RGB and depth features helps stabilize multimodal fusion. Because its effect is smaller than that of the reconstruction losses, this term acts as a regularizer rather than the main source of performance improvement.

**Figure 7:**
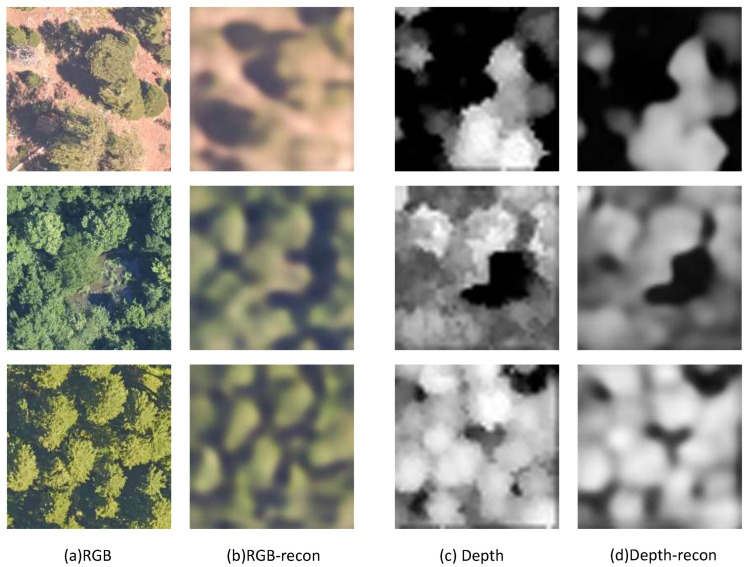
Visual examples of auxiliary reconstruction on the NEONTreeEvaluation test set. Each row shows the input RGB image, reconstructed RGB image, input depth map, and reconstructed depth map.

To further examine whether the auxiliary reconstruction branch provides a useful regularization signal for modality-aware feature learning, we visualize several reconstruction examples in Figure 7 and report the reconstruction error statistics in Table 13. Specifically, reconstruction quality is evaluated using MSE, MAE, peak signal-to-noise ratio (PSNR), structural similarity index measure (SSIM) [52], and gradient error (Grad. error). PSNR measures pixel-level reconstruction fidelity in decibels (dB), SSIM evaluates structural similarity, and Grad. error measures the discrepancy between image gradients, which reflects edge and local structure preservation. Higher PSNR and SSIM indicate better reconstruction quality, while lower MSE, MAE, and Grad. error indicate better performance. The gradient error corresponds to the gradient consistency term $L_{\mathrm{grad}}$ in the depth reconstruction loss and is defined as

(28) $$L_{\mathrm{grad}}={∥\nabla_{x}{\hat{I}}_{d}-\nabla_{x}I_{d}∥}_{1}+{∥\nabla_{y}{\hat{I}}_{d}-\nabla_{y}I_{d}∥}_{1},$$

where $\nabla_{x}$ and $\nabla_{y}$ denote the horizontal and vertical finite-difference gradient operators, respectively, and ${\hat{I}}_{d}$ and $I_{d}$ denote the reconstructed and input depth maps.

The reconstructed RGB images preserve the coarse color distribution and canopy layout of the input RGB images, while the reconstructed depth maps retain the main height-structure patterns of tree crowns and background regions. The reconstructed outputs are smoother than the original inputs, which is expected because they are generated from the fused latent representation and are used as training-time regularization rather than as final inference outputs.

As shown in Figure 7, the auxiliary decoders recover the main spatial structure of both modalities, although fine-grained texture details are smoothed. This behavior is also reflected in Table 13. The RGB reconstruction has an MSE of 0.018 and a PSNR of 17.79 dB, indicating that the reconstructed RGB branch retains coarse appearance information rather than producing pixel-level high-fidelity restoration. The depth reconstruction obtains an MAE of 0.058, an SSIM of 0.574, and a Grad. error of 0.009 suggesting that the height-related structure and local spatial variations are partially preserved. Overall, these results support the role of reconstruction supervision as an auxiliary regularizer that encourages the fused latent representation to encode task-relevant appearance and structural cues. Although the reconstructed RGB and depth maps are smoother than the original observations, they provide sufficient supervision for modality-aware feature learning.

A further comparison with the BM-style training method supports the proposed redesign of the first training stage. As shown in Table 14, the BM-style training variant performs substantially worse than the proposed reconstruction-oriented two-stage strategy, with the MAE increasing from 7.92 to 12.30 and the RMSE increasing from 14.20 to 20.10. These observations suggest that, for RGB–D tree counting, learning from self-reconstruction provides a more task-consistent initialization than directly imitating externally generated fused images.

Additional analysis on supervision form and kernel function. Finally, we examine the effect of the density supervision design by conducting controlled experiments on the full model. In these experiments, all model components and training configurations are kept unchanged, and only the construction of the density supervision target is modified. The corresponding results are reported in Table 15.

First, switching from bounding-box-guided supervision to point-guided supervision leads to degradation on all three metrics, with the MAE increasing from 7.92 to 8.71, the RMSE increasing from 14.20 to 16.01, and $R^{2}$ decreasing from 0.8684 to 0.8328. This result indicates that bounding-box-guided density supervision contributes positively to the final performance of BCAR-Net. A plausible explanation is that point supervision only provides object-center information, whereas bounding boxes additionally encode the coarse spatial extent of tree crowns. Such spatial cues are particularly useful in aerial forest scenes, where crowns often exhibit overlap, scale variation, and ambiguous boundaries.

We further evaluate the effect of different kernel functions for constructing the box-derived density target $\rho_{j}\left(x,y\right)$. Although both uniform and Gaussian forms are possible, the Gaussian box-density target was adopted in all main experiments. Compared with the uniform box target, the Gaussian target reduces the MAE from 8.62 to 7.92 and the RMSE from 15.44 to 14.20, while increasing $R^{2}$ from 0.8445 to 0.8684. This suggests that a center-weighted Gaussian target provides a more informative supervision signal than uniformly distributing the density mass over the whole bounding box. In overlapping canopy regions, a uniform target may assign similar weights to crown centers, crown boundaries, and background pixels enclosed by the box, which can blur the distinction between adjacent trees. In contrast, the Gaussian target concentrates larger density responses near the box center and assigns smaller weights near the boundary, helping the model learn more localized density responses around individual tree crowns.

### 3.6. Discussion

Structured multimodal fusion and failure cases. The comparative experiments and modality ablations indicate that BCAR-Net benefits from combining RGB appearance with height-related structural information. Direct RGB–D concatenation already improves over either single modality, while the broker representation, BCA-UNet interaction, and spatial fusion gate provide additional gains (Table 7, Table 8, Table 9 and Table 10). These results suggest that tree counting in aerial forest imagery requires not only access to geometric cues but also an effective way to align them with RGB texture and canopy appearance. The auxiliary reconstruction branch further provides training-time regularization for the fused representation, but its role should be understood as a representation constraint rather than as high-fidelity RGB or depth generation.

Nevertheless, the grouped error analysis shows that this fusion strategy is not uniformly effective under all forest conditions. As shown in Table 6, the largest errors are concentrated in high-density and high-canopy-closure patches, and both groups exhibit negative bias, indicating systematic under-counting in crowded and closed-canopy scenes. Such errors are closely related to the vertical and horizontal complexity of forest structure. In multi-layered stands, understory trees are often occluded by dominant canopy layers in nadir-view aerial imagery and may have weak or missing responses in both RGB imagery and CHM-derived height maps. High-density patches further increase the chance that several adjacent crowns are represented as a continuous canopy texture, while high-canopy-closure patches reduce the separability of individual crowns because both RGB boundaries and height discontinuities become less distinct. Overlapping crowns introduce a similar ambiguity: although the annotations may mark multiple neighboring trees, their RGB textures and height responses can appear as a single connected canopy region. Density regression avoids the need for explicit crown delineation, but it can still underestimate tree abundance when several crowns form a continuous texture or height pattern.

Data quality and forest condition also affect the reliability of the multimodal cues. CHM may vary across sites because of differences in point-cloud density, interpolation artifacts, terrain normalization errors, shadows, or missing values. When the height product is inaccurate or overly smoothed, the benefit of RGB–Depth fusion can be weakened. Dead trees, leaf-off trees, and sparse crowns further increase uncertainty because their visual appearance and height responses may differ from those of healthy broadleaf or conifer crowns. These factors are particularly relevant for cross-site evaluation, where species composition, phenological stage, illumination, crown morphology, and forest management history can vary substantially.

Practical use and deployment feasibility. From a practical perspective, BCAR-Net is more suitable for offline or near-offline patch-level and plot-level tree-count estimation where paired RGB and CHM are available. The method predicts density maps and obtains the final count by density integration; it does not explicitly output tree centers, crown boundaries, or instance-level boxes. Therefore, it should not be viewed as a replacement for individual tree detection or crown delineation methods when precise tree locations, crown extents, or tree-level attributes are required. The YOLO-based baseline provides a complementary detection-based reference, whereas BCAR-Net focuses on count estimation through density map regression. For deployment, the auxiliary reconstruction decoders are used only during training and do not increase the inference-stage parameter count. Even so, BCAR-Net still requires paired RGB and height inputs and contains a multimodal broker-fusion pipeline, which makes it more suitable for offline or near-offline monitoring than for strict real-time embedded deployment. In UAV or edge-computing scenarios, the computational cost of RGB–Depth alignment, height-map generation, and multimodal inference should be considered together. Future work will focus on lightweight backbones, cross-attention pruning, quantization, and knowledge distillation to improve its applicability to real-time UAV monitoring and resource-constrained forest assessment.

## 4. Conclusions

This study proposed BCAR-Net, a broker-guided RGB–D multimodal framework for tree counting in complex forest scenes, built upon four coordinated components within a unified density-regression pipeline: a bidirectional cross-attention U-Net that generates a broker RGB–D representation through symmetric RGB-Depth interaction and direction-aware gating, a tri-branch encoder with a spatial fusion gate that adaptively aggregates RGB, depth, and broker features, a multi-scale cross-attention reconstruction decoder coupled with a reconstruction-oriented two-stage training scheme that replaces externally generated fused-image supervision with task-consistent self-reconstruction, and a bounding-box-guided density supervision tailored to the spatial extent of tree crowns. Experiments on the NEONTreeEvaluation test set and a public UAV RGB-LiDAR test set showed that BCAR-Net consistently outperformed single-modality baselines, direct RGB-D concatenation, a YOLO-based detection baseline, and a transferred BM-style multimodal baseline, with ablation studies confirming that the gains stem from the coordinated effect of all four designs rather than any single component. At the same time, the method still involves relatively high computational cost and requires paired RGB and CHM inputs; therefore, it is currently more suitable for offline or near-offline patch-level and plot-level tree-count estimation than for strict real-time embedded deployment. Taken together, these results show that BCAR-Net improves density map-based tree-count estimation on the tested NEONTreeEvaluation and UAV RGB–LiDAR datasets when paired RGB imagery and height products are available. Future work should also explore lightweight backbones, attention pruning, quantization, and knowledge distillation to improve the applicability of the model in UAV and edge-computing scenarios. 

## Figures and Tables

**Table 1:** Site-level composition of the retained NEONTreeEvaluation samples after preprocessing.

Site Group I				Site Group II			
**NEON Site**	**Train**	**Test**	**Total**	**NEON Site**	**Train**	**Test**	**Total**
ABBY	0	2	2	NIWO	18	11	29
BART	0	2	2	OSBS	178	14	192
BLAN	0	2	2	SCBI	0	2	2
BONA	0	4	4	SERC	0	2	2
CLBJ	0	2	2	SJER	507	61	568
DELA	1	2	3	SOAP	0	2	2
DSNY	0	6	6	TALL	0	2	2
HARV	0	3	3	TEAK	64	51	115
JERC	0	6	6	TOOL	169	0	169
LENO	0	2	2	WREF	0	5	5
MLBS	0	8	8				
Total	937	189	1126				

**Table 2:** Summary of the data construction protocol used in this study. Native resolution or point density and approximate ground coverage are reported to clarify the pixel-level and metric scale of the input patches. Approximate ground coverage was calculated according to the RGB spatial resolution.

Dataset	Split	Site/Area	Source Data	Input Modalities	Native Resolution/Density	Pixel Patch Size/Ground Coverage	Samples
NEON	Training	6 NEON sites	Benchmark training subset	RGB + CHM/LiDAR-derived depth	RGB: 0.1m/pixel; CHM raster: ∼1m, resampled to RGB grid	1024×1024 px; ∼102.4m×102.4m	937
	Testing	20 NEON sites	Benchmark evaluation subset			400×400 px; ∼40m×40m	189
UAV	Training	Perm Krai, Russia	Public UAV RGB–LiDAR dataset	RGB + CHM-derived depth	RGB: 0.07m/pixel; LiDAR: $∼37\;\mathrm{pts}/m^{2}$	400×400 px; ∼28m×28m	72
	Testing						57

**Table 3:** Computational complexity comparison.

Model	Total Params (M)	Inference Params (M)	FLOPs (G)
P2RLoss	34	17	99
STEERER	65	65	105
BM	40	40	557
BCAR-Net	74	40	557

**Table 4:** Performance comparison with representative detection-based and density-regression-based tree counting methods on the NEONTreeEvaluation test set.

Input Type	Method	MAE ↓	RMSE ↓	$R^{2}$↑
RGB	SENet50	19.74	32.05	0.3496
	ResNet50	14.69	25.52	0.5876
	STEERER	12.09	21.28	0.7134
	YOLOv8	11.69	18.59	0.7811
Depth	APGCC	21.49	34.40	0.1763
	ResNet50	17.53	29.32	0.4405
	P2RLoss	14.21	29.95	0.4325
	STEERER	11.21	22.67	0.6747
RGB+Depth	BM	13.64	21.52	0.6979
	Ours	**7.92**	**14.20**	**0.8684**

↑ indicates higher is better; ↓ indicates lower is better.

**Table 5:** Performance comparison with representative tree counting methods on the UAV test set. Results are reported as mean ± standard deviation over repeated independent runs.

Method	MAE ↓	RMSE ↓	$R^{2}$↑
STEERER-RGB	23.14 ± 3.57	31.08 ± 5.74	−0.20 ± 0.44
STEERER-Depth	14.59 ± 1.47	18.53 ± 1.49	0.57 ± 0.06
P2RLoss-Depth	12.38 ± 0.33	15.10 ± 0.38	0.72 ± 0.01
Ours	**11.56 ± 0.32**	**14.70 ± 0.33**	**0.7360 ± 0.0127**

↑ indicates higher is better; ↓ indicates lower is better.

**Table 6:** Systematic error analysis on the NEONTreeEvaluation test set. Tree density, crown size, and canopy closure groups were defined by tertiles on the test set. Bias denotes the mean signed counting error.

Factor	Group	Samples	MAE ↓	RMSE ↓	$R^{2}$↑	Bias
Tree density	Low	68	1.84	2.75	−0.2954	1.27
	Medium	59	5.19	8.61	0.1477	0.39
	High	62	17.19	23.15	0.7309	−9.21
Crown size	Small	63	12.96	20.50	0.8349	−8.90
	Medium	63	8.08	11.89	0.6417	0.04
	Large	63	2.72	6.58	−0.4665	1.53
Canopy closure	Low	63	2.42	4.29	0.9211	0.32
	Medium	63	4.17	5.84	0.9383	−1.47
	High	63	17.17	23.50	0.7566	−6.17
Site/area	SJER	61	1.85	2.55	0.6822	0.38
	TEAK	51	4.75	6.14	0.9076	−1.12
	OSBS	14	8.29	12.46	0.6619	−7.50
	NIWO	11	24.21	35.89	0.6849	−17.86
	Other sites	52	14.60	19.34	0.1892	−2.43

↑ indicates higher is better; ↓ indicates lower is better.

**Table 7:** Effect of input modality and broker RGB–D representation on the NEONTreeEvaluation test set. Results are reported as mean ± standard deviation over repeated independent runs.

Input Setting	MAE ↓	RMSE ↓	$R^{2}$↑
RGB	11.37 ± 0.09	18.36 ± 0.01	0.77 ± 0.01
Depth	10.34 ± 0.13	19.36 ± 0.23	0.75 ± 0.01
RGB–D (simple concatenation)	8.87 ± 0.21	15.79 ± 0.23	0.83 ± 0.011
RGB–D (proposed)	**8.18 ± 0.22**	**14.52 ± 0.35**	**0.86 ± 0.01**

↑ indicates higher is better; ↓ indicates lower is better.

**Table 8:** Effect of bidirectional cross-attention in BCA-UNet on the NEONTreeEvaluation test set. Results are reported as single-run controlled comparisons.

Configuration	MAE ↓	RMSE ↓	$R^{2}$↑
Cross-Attn (BCA-UNet)	8.38	14.80	0.8571
Single Attn (BCA-UNet)	8.72	15.71	0.8390
Full Model	**7.92**	**14.20**	**0.8684**

↑ indicates higher is better; ↓ indicates lower is better.

**Table 9:** Effect of Transformer encoder depth in the tri-branch backbone on the NEONTreeEvaluation test set. Results are reported as single-run controlled comparisons.

Encoder Depth	MAE ↓	RMSE ↓	$R^{2}$↑
0 (CNN only)	9.11	16.25	0.8276
1	7.92	14.20	0.8684
2	9.05	15.75	0.8381
3	9.71	17.59	0.7981

↑ indicates higher is better; ↓ indicates lower is better.

**Table 10:** Effect of tri-branch adaptive fusion on the NEONTreeEvaluation test set. Results are reported as single-run controlled comparisons.

Configuration	MAE ↓	RMSE ↓	$R^{2}$↑
Spatial Fusion Gate	9.21	16.31	0.8263
Broker RGB–D Branch	8.23	14.40	0.8646
Full Model	7.92	14.20	0.8684

↑ indicates higher is better; ↓ indicates lower is better.

**Table 11:** Effect of multi-scale cross-attention reconstruction on the NEONTreeEvaluation test set. Results are reported as single-run controlled comparisons.

Configuration	MAE ↓	RMSE ↓	$R^{2}$↑
Cross-Attn (Decoder)	9.28	16.12	0.8305
Full Model	7.92	14.20	0.8684

↑ indicates higher is better; ↓ indicates lower is better.

**Table 12:** Effect of auxiliary supervision and regularization on the NEONTreeEvaluation test set. Results are reported as single-run controlled comparisons.

Training Setting	MAE ↓	RMSE ↓	$R^{2}$↑
× Total Reconstruction	8.98	16.53	0.8216
Depth Reconstruction	8.23	15.69	0.8394
RGB Reconstruction	8.04	15.39	0.8455
Feature Consistency	8.20	15.07	0.8517
Full Model	7.92	14.20	0.8684

↑ indicates higher is better; ↓ indicates lower is better.

**Table 13:** Reconstruction error statistics on the NEONTreeEvaluation test set. RGB and depth values were converted back to the normalized [0,1] range before metric calculation. PSNR is reported for RGB reconstruction, while Grad. error is reported for depth reconstruction.

Target	MSE ↓	MAE ↓	PSNR ↑	SSIM ↑	Grad. Error ↓
RGB	0.018	0.10	17.79	0.24	–
Depth	0.007	0.058	–	0.574	0.009

↑ indicates higher is better; ↓ indicates lower is better.

**Table 14:** Effect of two-stage training strategy on the NEONTreeEvaluation test set. Results are reported as single-run controlled comparisons.

Training Strategy	MAE ↓	RMSE ↓	$R^{2}$↑
BM-style Training	12.30	20.10	0.7364
Ours	7.92	14.20	0.8684

↑ indicates higher is better; ↓ indicates lower is better.

**Table 15:** Effect of supervision form and kernel function on the NEONTreeEvaluation test set. Results are reported as single-run controlled comparisons.

Supervision Type	MAE ↓	RMSE ↓	$R^{2}$↑
Point-guided density supervision	8.71	16.01	0.8328
Bounding-box-guided supervision, uniform target	8.62	15.44	0.8445
Bounding-box-guided supervision, Gaussian target	7.92	14.20	0.8684

↑ indicates higher is better; ↓ indicates lower is better.

## Data Availability

The datasets used in this study are publicly available and can be downloaded from https://github.com/weecology/NeonTreeEvaluation and https://www.kaggle.com/datasets/sentinel3734/tree-detection-lidar-rgb. Users are requested to cite the relevant dataset publications when using these datasets. The source code for BCAR-Net can be accessed at https://github.com/wxy-cocoa/BCAR-Net, all websites accessed in 28 May 2026.
